# Adipokines as Potential Biomarkers in Rheumatoid Arthritis

**DOI:** 10.1155/2014/425068

**Published:** 2014-03-30

**Authors:** Annalisa Del Prete, Valentina Salvi, Silvano Sozzani

**Affiliations:** ^1^Department of Molecular and Translational Medicine, University of Brescia, Viale Europa 11, 25123 Brescia, Italy; ^2^Humanitas Clinical and Research Center, via Manzoni 113, 20089 Rozzano, Italy

## Abstract

Rheumatoid arthritis (RA) is a chronic systemic inflammatory autoimmune disease characterized by severe joint injury. Recently, research has been focusing on the possible identification of predictor markers of disease onset and/or progression, of joint damage, and of therapeutic response. Recent findings have uncovered the role of white adipose tissue as a pleiotropic organ not only specialized in endocrine functions but also able to control multiple physiopathological processes, including inflammation. Adipokines are a family of soluble mediators secreted by white adipose tissue endowed with a wide spectrum of actions. This review will focus on the recent advances on the role of the adipokine network in the pathogenesis of RA. A particular attention will be devoted to the action of these proteins on RA effector cells, and on the possibility to use circulating levels of adipokines as potential biomarkers of disease activity and therapeutic response.

## 1. Introduction

An emerging body of evidence suggests that the white adipose tissue (WAT) plays more than just the role of energy storage compartment and thermal and mechanical insulator. WAT is now recognized as a pleiotropic organ specialized in endocrine functions being able to produce several hormones and other proteins involved in both physiological and pathological processes, including immunity and inflammation [1]. The biological active substances secreted by WAT contribute to the systemic “low-grade inflammatory state” associated with obesity [2, 3]. Indeed, increased circulating levels of several markers of inflammation occur in obese subjects, such as IL-6, TNF-*α*, C-reactive protein (CRP), and plasminogen activator inhibitor I (PAI-I) [4, 5]. It should be also considered that infiltrating macrophages represent an important source of inflammatory mediators which further promote and sustain inflammation [6]. The term “adipokines” is applied to all the biological active substances synthesized by WAT which function as regulators of energy homeostasis and metabolism; the same mediators are also involved in chronic inflammation and metabolic dysfunctions [7].

Rheumatoid arthritis (RA) is a chronic systemic autoimmune disorder characterized by synovial inflammation, cartilage damage, and bone erosion, with 1% prevalence worldwide [8]. Although the pathogenesis of this disease is poorly understood, several observations indicate that adipokines affect tissues and cells involved in RA, including synovium, cartilage, bone, and immune cells [9]. In the present review we will describe the information available on the role of adipokines in RA pathogenesis, focusing on the role of adiponectin, leptin, chemerin, visfatin, resistin, lipocalin 2, SAA3, and a few others, in light of their possible consideration as new potential circulating biomarkers of disease activity and therapeutic response.

## 2. Adiponectin

Adiponectin (also called GBP28, AdipoQ, ApM1, and Acrp30) is a collagen-like protein with a structure similar to the complement factor C1q. Adiponectin is mainly produced by adipocytes and is present, in different molecular isoforms, at high levels (3–30 *μ*g/mL) in the blood [10, 11]. Two adipokine receptors were recently identified, AdipoR1, mainly expressed in skeletal muscles, and AdipoR2 which is expressed in the liver [12]. The signaling transduction pathways of adiponectin receptors involve the activation of the adaptor protein APLL1 [13] and many signaling molecules, including AMPK, p38 MAP kinases, and PPAR-*α* and PPAR-*γ* [10, 14]. The main functions of adiponectin are, in the muscle, the increase of fatty acid oxidation and glucose uptake and, in the liver, the reduction of glucose synthesis.

**Figure 1:**
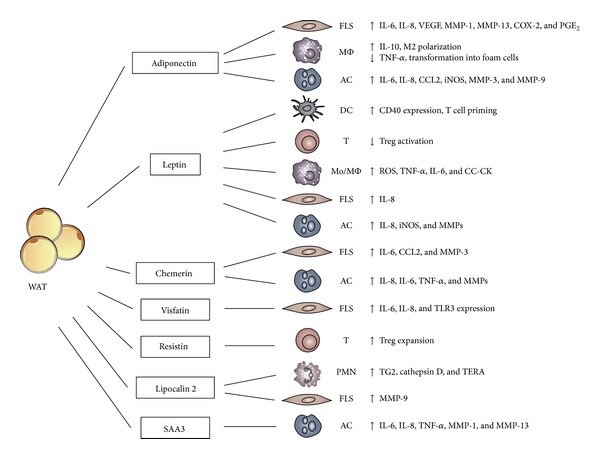
Role of adipokines on RA effector cells. The role of different adipokines on RA target cells is illustrated in the figure. WAT: white adipose tissue, SAA3: serum amyloid A3, FLS: fibroblast-like synoviocytes, AC: articular chondrocytes, PMN: neutrophils, MMP: metalloprotease, COX-2: cyclooxygenase 2, ROS: reactive oxygen species, iNOS: inducible nitric oxide synthase, CC-CK: CC-chemokines, TG2: transglutaminase 2, and TERA: transitional endoplasmic reticulum ATPase.

Low levels of circulating adiponectin, as those observed in obesity, type 2 diabetes, atherosclerosis, vessel inflammation, and metabolic syndrome, suggest a protective function. Accumulating evidence supports a potential role of adiponectin in controlling inflammation. For instance, adiponectin was reported to inhibit the transformation of macrophages into foam cells [15], to stimulate the production of the anti-inflammatory cytokine IL-10 [16], to reduce the production of TNF-*α* [17], to induce tolerance in response to TLR ligands [18], and to promote the anti-inflammatory M2 macrophage polarization (Figure 1) [19]. The anti-inflammatory effects of adiponectin have been, to some extent, ascribed to its capacity to alter ceramide metabolism and to promote sphingosine-1-phosphate synthesis [20]. However, evidence that adiponectin may act as a proinflammatory mediator promoting extracellular matrix degradation and joint disruption is also available. Indeed, in cultured chondrocytes, adiponectin increases the expression of MMP-3 [21] and the secretion and activity of proinflammatory mediators, such as nitric oxide synthase type II (NOS2/iNOS), MMP-9, IL-6, MCP-1, and IL-8 [22, 23]. Similarly, adiponectin is able to stimulate the production of PGE2, IL-6, IL-8, vascular endothelial growth factor (VEGF), MMP-1 and MMP-13, cyclooxygenase 2 (COX-2), and microsomal prostaglandin E synthase 1 (mPGES-1) [24, 25] in RA synovial fibroblasts (Figure 1). In RA, the cellular targets of adiponectin may also include lymphocytes and endothelial cells, further supporting the role of adiponectin in this pathology [26].

In RA patients, the serum/plasma levels of adiponectin, as well as the levels in the synovial fluid, are associated with radiographic damage [27] and are increased compared to osteoarthritis patients (OA) and healthy donors [28, 29]. Increased adiponectin levels positively correlate with the disease activity score 28 (DAS28), the erythrocyte sedimentation rate (ESR), and the rheumatoid factor (RF) [30]. Recently, Klein-Wieringa et al. reported that the baseline levels of adiponectin can also predict radiographic progression over a four-year period independently of the presence of anticyclic citrullinated peptide (CCP) antibodies and body mass index (BMI) [31]. In addition, the elevation of total and high molecular weight adiponectin was described in patients with RA treated with anti-TNF agents (e.g., infliximab and etanercept) [32, 33] (Table 1). Finally, considering the detrimental effects of this adipokine in perpetuating joint inflammation, the use of adiponectin as a potential therapeutic target of blocking therapies has been proposed [34].

## 3. Leptin

Leptin, the product of* ob* gene, is a 16 kDa nonglycosylated hormone peptide [35] which binds the OB-Rb long form leptin receptor coupled to a JAK/STAT signaling pathway [36, 37]. Leptin is considered the major regulator of body weight, since it induces the decrease of food intake and increases energy consumption [38]. Leptin is mainly produced by WAT and the circulating levels of leptin correlate positively with the amount of adipose tissue and BMI [39]. However, leptin synthesis is also regulated by the action of inflammatory mediators [40]. Leptin is generally considered a proinflammatory adipokine. In fact, leptin stimulates the production of proinflammatory cytokines, such as TNF-*α* and IL-6, and reactive oxygen species in cultured monocytes. In addition, it induces the production of CC-chemokines by macrophages and alters the Th1/Th2 balance favoring the Th1 phenotype (Figure 1) [41–43]. Moreover, leptin null mice are protected in experimental models of T cell mediated hepatitis and experimental autoimmune encephalomyelitis [44, 45].

Leptin has been associated with autoimmune diseases, in particular with RA. However, there are conflicting observations concerning the circulating levels of leptin in RA patients, since some studies suggested a correlation between leptin levels and disease activity [28, 46, 47], while others failed to detect changes in circulating leptin levels [48]; interference of concomitant pharmacological treatments might be responsible for these apparently contrasting results. In experimental models of arthritis, leptin deficient mice showed a milder form of antigen-induced arthritis associated with the reduction of IFN-*γ* production and the increase in IL-10 secretion by in vitro reactivated lymph node cells [49]. In contrast, leptin-deficient and leptin receptor-deficient mice exhibited a delayed resolution of the disease [50]; the administration of leptin ameliorated disease activity [51]. These conflicting results do not allow coming to a clear conclusion on the role of leptin in RA. To note, leptin circulating levels apparently are not modulated in patients treated with anti-TNF-*α* therapy [52–54] (Table 1). Recently, the serum/synovial fluid ratios of leptin levels were associated with disease duration and erosion [55]. In addition, several in vitro studies sustained the pathogenic role of leptin in RA. In human and murine chondrocytes, leptin synergizes with IL-1*β* and IFN*γ* for the activation of type 2 nitric oxide synthase (NOS) and the induction of IL-8 and metalloproteinases via a JAK2, PI3K, and MAP kinase-dependent signaling pathway [23, 56–58]. Leptin also induced IL-8 in human synovial fibroblasts with a NF*κ*B-dependent pathway [59]. Furthermore, leptin can also modulate the activities of several immune cells [60]. For instance, in murine dendritic cells, leptin increases CD40 expression and T cell priming (Figure 1) [61]. Matarese et al. showed that leptin-null and leptin receptor-null mice have increased levels of Treg cells and are protected in experimental models of autoimmune diseases [45]. In keeping with this observation, high leptin levels are associated with a reduction of Treg and with the activation of proinflammatory effector T cells [62–64]. Recently, it was shown that the leptin-induced state of overexpression of the mTOR pathway, in freshly isolated Treg cells, is responsible for their state of hyporesponsiveness. Therefore, it is conceivable that Treg activation is dependent on the dynamic regulation of mTOR activity by the composition of the extracellular milieu, such as the concentrations of leptin and cell nutrients [65]. These results clearly depict leptin as a pleiotropic molecule placed at the crossroads of immune tolerance, metabolism, and autoimmunity. Further studies are needed in order to clarify whether leptin might represent a new disease activity biomarker and to explore its therapeutic potential in autoimmune diseases.

## 4. Chemerin

Chemerin is a 16 kDa protein, originally described as the product of the Tazarotene-induced gene 2 (Tig2) [66] and purified from ascitic fluids of ovarian cancer patients and synovial exudates of rheumatoid arthritis patients [67]. Chemerin is secreted as an inactive precursor protein which is subsequently converted into a bioactive protein following the proteolytic removal of the last six or seven amino acids from the C-terminal end [68]. Chemerin was first described as the functional ligand of the chemotactic receptor ChemR23. Dendritic cells, macrophages, and NK cells express ChemR23 and a role for chemerin in their recruitment into inflammatory sites was described in lupus erythematosus, oral lichen planus, and psoriasis [69–72]. More recently, the adipokine function of chemerin was proposed, since chemerin is mainly produced by WAT and plays important regulatory role in adipogenesis in vitro [73]. In addition, chemerin is considered a biomarker of adiposity, because chemerin levels strongly associate with BMI [74], markers of inflammation (e.g., TNF-*α*, IL-6, and CRP) [75], and metabolic syndrome [76]; chemerin circulating levels decrease with weight and fat loss [77]. Human articular chondrocytes express chemerin and its receptor ChemR23 and secrete proinflammatory cytokines, such as IL-6, IL-8, and TNF-a, and metalloproteases, in response to chemerin stimulation (Figure 1) [78]. In RA patients the expression of chemerin and ChemR23 in fibroblast-like synoviocytes (FLS) was found increased compared to OA patients. Chemerin was reported to mediate direct proinflammatory and stimulatory effects on the RA-FLS [79], suggesting a pivotal role of the chemerin/ChemR23 axis in the pathogenesis of RA. A recent study reported that RA patients have increased levels of circulating chemerin and chemerin levels positively correlated with disease activity (DAS28, ESR, and CRP) [80]. Circulating chemerin levels are negatively regulated by the anti-TNF therapy (adalimumab) in parallel with the reduction of disease activity markers, such as DAS28, ESR and CRP, and IL-6, and the macrophage migration inhibitory factor (MIF) levels [81] (Table 1). These results nominate chemerin serum levels as a biomarker for disease activity and therapeutic response.

## 5. Visfatin

Visfatin, also known as pre-B-cell colony-enhancing factor (PBEF) and nicotinamide phosphoribosyltransferase (Nampt), was originally described as a cytokine involved in early B-cell development and was later renamed visfatin since it is secreted mainly by visceral fat [82]. In addition, leukocytes, in particular granulocytes and monocytes/macrophages, from obese patients produce high levels of visfatin [83–85]. Visfatin is also produced by endotoxin-challenged neutrophils, where it functions as an antiapoptotic molecule acting at level of caspases 3 and 8 [86]. Visfatin was also suggested to have insulin-like functions [87, 88]. A specific receptor for visfatin has not been identified yet. Nevertheless, the proinflammatory action of visfatin was described to be mediated by the insulin signaling pathway through Akt phosphorylation [89].

Circulating levels of visfatin correlate with obesity and type 2 diabetes and are reduced after weight loss [90]. Visfatin was also proposed to promote atherosclerosis and to cause plaque destabilization through the induction of proinflammatory mediators and adhesion molecules in endothelial cells [91–93]. Several observations sustain the hypothesis that visfatin may play a major role in the pathogenesis of RA. Recent studies reported the upregulation of visfatin in activated RA-SFs in response to proinflammatory stimuli, such as IL-6 and the activation of TLR3 [94, 95] with visfatin acting as an autocrine positive feedback mechanism for IL-6 production [96]. In RA synovium, visfatin was predominantly expressed in the lining layer, lymphoid aggregates, and interstitial vessels. In RA-SFs, visfatin induced high amounts of chemokines such as IL-8 and CCL2, proinflammatory cytokines (i.e., IL-6), and matrix metalloproteinases (i.e., MMP-3) (Figure 1). Visfatin promoted fibroblast migration and induced phosphorylation of p38 MAPK; of note, inhibition of p38MAPK strongly reduced visfatin effects [97]. Finally, visfatin inhibition significantly reduced the severity of the disease and TNF-*α* circulating levels in the experimental model of collagen-induced arthritis [98, 99].

In RA, circulating levels of visfatin are increased [28], as well as its expression in synovial fluids and inflamed synovium [94–96]. Visfatin serum and synovial fluid levels correlated with the degree of inflammation, with the severity of the disease, and with joint damage [31, 95, 100]. Contradictory results are available on visfatin levels in patients undergoing anti-TNF-*α* therapy. In one study no significant changes were observed [101], while in others a negative correlation with therapy was found [91]. In general, visfatin serum levels better correlated with the number of circulating B cells rather than with the disease activity and were profoundly affected after B-cell depletion therapy with rituximab. The lack of change in serum visfatin levels is suggested to predict worsening disease activity [102] (Table 1).

## 6. Resistin

Resistin is a cysteine-rich protein of 12.5 kDa also known as adipocyte-secreted factor (ASF) or “found in inflammatory zone 3” (FIZZ3) [103]. In RA experimental models, resistin promotes insulin resistance, while the function in humans is still unclear [104]. Even if resistin was originally described to be produced only by WAT, subsequent studies demonstrated that, in humans, resistin mainly derives from circulating monocytes and macrophages [105]. The resistin receptor is still unknown and recently TLR4 was proposed to mediate resistin proinflammatory functions in human cells [106]. Resistin has a strong impact on immune functions. It can enhance the expansion of Treg cells through an effect on dendritic cells (Figure 1) [107]. Proinflammatory mediators increase resistin expression; in turn, resistin induces TNF-*α*, IL-12, IL-6, and IL-1*β* production [108, 109]. These findings, together with the observation that the intra-articular injection of resistin in the knee joints induces arthritis, sustain the involvement of resistin in RA pathogenesis [110]. Several reports have demonstrated that serum resistin levels are significantly higher in RA than in OA patients or healthy controls [111–113]. The increased serum levels of resistin correlated with markers of inflammation, such as CRP, ESR, IL-1Ra, and total leukocyte count [47, 114–117], disease activity (DAS28), and joint destruction [112]. However, these results were not confirmed by other groups [111], and conflicting results were reported on the association between resistin and radiographic progression signs [27, 31, 100]. Recently, the anti-TNF-*α* therapy was reported to modulate resistin levels in RA patients [118, 119] (Table 1). Resistin levels in synovial fluids and in the sublining layer are higher in RA than in OA patients [29, 110, 112]. These results strongly suggest that resistin production is elevated at the site of inflammation and accumulates in the synovial fluid of RA patients. In anti-CCP positive patients, synovial fluid resistin levels, but not serum levels, correlated with disease progression suggesting resistin as a disease progression marker [111].

## 7. Lipocalin 2

Lipocalin 2 (LCN2), also known as siderocalin, 24p3, uterocalin, and neutrophil gelatinase-associated lipocalin (NGAL), is a recently identified glycoprotein stored in neutrophil granules [120] but mainly produced by WAT [121, 122]. LCN2 has been isolated in different isoforms and its functions are carried out by the activation of the cellular receptor megalin [123]. LCN2 binds and transports small lipophilic substances, such as retinoids, arachidonic acid, steroids, iron, and fatty acids [124–126]. Other functions that have been attributed to LCN2 are the induction of apoptosis in hematopoietic cells [127], the inhibition of bacterial growth [128, 129], regulation of iron metabolism [130], and insulin resistance [131]. LCN2 is induced by inflammatory stimuli through the activation of the NFkB pathway [132]; however dexamethasone promotes LCN2 production in chondrocytes [133, 134]. LCN2 is involved in the allosteric activation of MMP-9 [135] and levels of MMP-9 are higher in the serum and synovial fluid of RA patients [136]. Recently, LCN2 synovial fluid levels were found to be increased in RA compared to OA patients [137]. Through a proteomic approach, GM-CSF was found to induce LNC2 upregulation in neutrophils, which in turn can influence synoviocyte behavior through the release of several enzymes, such as transglutaminase 2 (TG2), cathepsin D, and transitional endoplasmic reticulum ATPase (TERA) (Figure 1), which contribute to both inflammation of synovium and proliferation of synovial cells, promoting the RA state [137].

## 8. SAA3

The serum amyloid A3 (SAA3) belongs to the family of acute phase serum amyloid A proteins produced by hepatocytes [138] and other cell types, including adipocytes [139, 140]. SAA3 was associated to altered metabolic and immunocompromised conditions [141, 142]. Several stimuli, such as TNF-*α*, IL-1*β*, dexamethasone, IL-6, and LPS, can increase SAA3 expression [139, 140, 143]. Recently, SAA3 was suggested to directly activate the MyD88-dependent TLR4/MD-2 pathway [144].

In a rabbit Ag-induced arthritis model, upregulation of SAA3 transcripts was detected in cells infiltrating into the inflamed joint, in the area where pannus formation starts and, most notably, also in chondrocytes. In vitro, recombinant human SAA induces matrix metalloproteinase transcription in human chondrocytes (Figure 1). Further, SAA is highly expressed in human RA synovium [145]. Recently, Geurts et al. proposed that a SAA3-promoter report may have a diagnostic value in the classification of RA molecularly distinct forms with different degree of synovial tissue inflammation [146].

## 9. Other Adipokines

Vaspin, visceral adipose tissue-derived serine protease inhibitor, is expressed predominantly in visceral adipose tissue [147]. Expression of the vaspin gene positively correlates with BMI and administration of the protein to obese mice improved glucose tolerance and insulin sensitivity [147, 148]. Vaspin levels are increased in the serum and synovial fluid of RA patients [149, 150] (Table 1).

Omentin, also known as intelectin, is a protein secreted by omental adipose tissue and highly abundant in human plasma [151]. Both circulating protein levels and mRNA levels in adipose tissue decrease in obese subjects and correlate negatively with markers of obesity, such as BMI, waist circumference, and circulating leptin [152] (Table 1). Expression of the omentin gene was reported in omental adipose tissue of patients with Crohn's disease, suggesting a role in chronic inflammatory diseases [151]. The levels of omentin were found significantly reduced in the synovial fluid of patients with RA compared to OA patients [150]. On the contrary, circulating levels of omentin were significantly higher in patients with juvenile idiopathic arthritis compared to healthy controls [153].

Apelin is a bioactive peptide, originally identified as the endogenous ligand of the G-protein coupled receptor APJ [154]. Apelin is mainly produced by adipocytes, its expression is upregulated by insulin, and TNF-*α* and its levels are increased in obesity [155, 156]. Apelin has been implicated in the pathogenesis of OA, since high circulating levels are increased in the sera and synovial fluids of OA patients [157]. In early-stage RA patients serum apelin levels were found to be decreased but were insensitive to pharmacological treatment [158] (Table 1).

Adipsin, also known as complement factor D, is highly expressed in adipose tissue and in activated monocyte/macrophages [159]. Circulating levels of adipsin did not predict the radiographic progression of early-stage disease [31]; however, increased adipsin levels were found to be associated with a higher remission rate in early RA patients treated with DMARD [160] (Table 1).

## 10. Conclusions

The discovery of adipokines has profoundly changed our understanding of the functions of adipose tissue. The adipokine network is involved in the interplay between WAT, metabolic disorders, and immune-mediated diseases. Adipokines have shown to be able to modulate several aspects of inflammation as well as both innate and adaptive immune responses. Although in the past few years the implications of the adipokines in autoimmune diseases, including rheumatoid arthritis, have greatly increased, a clear picture of the role of these proteins in the pathogenesis and in the progression of this disease is still missing. Nevertheless, accumulating evidence on the modulation of serum and synovial fluid levels of many adipokines encourages their future exploitation as soluble biomarkers of disease activity and therapeutic response. Further studies are needed in order to translate the increasing number of experimental and clinical observations to the use of adipokines as clinical diagnostic markers.

## Figures and Tables

**Table 1:** Correlation of adipokines with disease activity parameters and therapeutic response.

Adipokine	Correlation with					
	DAS28	BMI	IL-6/TNF/ESR	Anti-CCP	Radiographic progression	Therapeutic response
Adiponectin	pos [30]	neg [31]	pos [30]	neg [31]	pos [27, 31]	Anti-TNF: pos [32, 33]
Leptin	pos [46]	neg [46]	neg [31]	no [31]	neg [98]	Anti-TNF: neg [52]
Chemerin	pos [80]	neg, pos [80]	pos [78]	ND	ND	Anti-TNF: pos [81]
Visfatin	neg [102]	neg [149]	pos [31]	pos [31], neg [102]	pos [100]	Anti-TNF: neg [101], pos [91] Anti-CD20: pos [102]
Resistin	pos [30]	pos [31]	pos [31, 118]	No in serum but pos in SF [111]	ND	Anti-TNF: pos [118]
Lipocalin 2	ND	ND	ND	ND	ND	ND
SAA3	ND	ND	ND	ND	ND	ND
Vaspin	SF pos [150]	pos [147]	SF neg [150]	SF neg [150]	ND	ND
Omentin	SF neg [150]	neg [152]	SF neg [150]	SF pos [150]	ND	ND
Apelin	ND	ND	ND	ND	ND	DMARDs: neg [158]
Adipsin	ND	pos [31]	pos [31]	ND	neg [31]	DMARDs: pos [160]

Abbreviations: pos: positive; neg: negative; SF: synovial fluid; ND: not determined. Where not specified, the correlations are referred to serum levels. Positive correlation with therapeutic response is assumed when the adipokine levels are modified (either they increase or decrease) by the treatment.

## References

[1] Hotamisligil GS (2006). Inflammation and metabolic disorders. *Nature*.

[2] Yudkin JS, Stehouwer CDA, Emeis JJ, Coppack SW (1999). C-reactive protein in healthy subjects: associations with obesity, insulin resistance, and endothelial dysfunction: a potential role for cytokines originating from adipose tissue?. *Arteriosclerosis, Thrombosis, and Vascular Biology*.

[3] Ouchi N, Kihara S, Funahashi T, Matsuzawa Y, Walsh K (2003). Obesity, adiponectin and vascular inflammatory disease. *Current Opinion in Lipidology*.

[4] Hotamisligil GS, Shargill NS, Spiegelman BM (1993). Adipose expression of tumor necrosis factor-*α*: direct role in obesity-linked insulin resistance. *Science*.

[5] Shimomura I, Funahashi T, Takahashi M (1996). Enhanced expression of PAI-1 in visceral fat: possible contributor to vascular disease in obesity. *Nature Medicine*.

[6] Weisberg SP, McCann D, Desai M, Rosenbaum M, Leibel RL, Ferrante AW (2003). Obesity is associated with macrophage accumulation in adipose tissue. *Journal of Clinical Investigation*.

[7] Ouchi N, Parker JL, Lugus JJ, Walsh K (2011). Adipokines in inflammation and metabolic disease. *Nature Reviews Immunology*.

[8] Bingham CO (2002). The pathogenesis of rheumatoid arthritis: pivotal cytokines involved in bone degradation and imflammation. *Journal of Rheumatology*.

[9] Gómez R, Conde J, Scotece M, Gómez-Reino JJ, Lago F, Gualillo O (2011). What’s new in our understanding of the role of adipokines in rheumatic diseases?. *Nature Reviews Rheumatology*.

[10] Kadowaki T, Yamauchi T (2005). Adiponectin and adiponectin receptors. *Endocrine Reviews*.

[11] Oh DK, Ciaraldi T, Henry RR (2007). Adiponectin in health and disease. *Diabetes, Obesity and Metabolism*.

[12] Yamauchi T, Nio Y, Maki T (2007). Targeted disruption of AdipoR1 and AdipoR2 causes abrogation of adiponectin binding and metabolic actions. *Nature Medicine*.

[13] Deepa SS, Dong LQ (2009). Appl1: role in adiponectin signaling and beyond. *American Journal of Physiology. Endocrinology and Metabolism*.

[14] Kadowaki T, Yamauchi T (2011). Adiponectin receptor signaling: a new layer to the current model. *Cell Metabolism*.

[15] Ouchi N, Kihara S, Arita Y (2001). Adipocyte-derived plasma protein, adiponectin, suppresses lipid accumulation and class A scavenger receptor expression in human monocyte-derived macrophages. *Circulation*.

[16] Kumada M, Kihara S, Ouchi N (2004). Adiponectin Specifically Increased Tissue Inhibitor of Metalloproteinase-1 Through Interleukin-10 Expression in Human Macrophages. *Circulation*.

[17] Yokota T, Oritani K, Takahashi I (2000). Adiponectin, a new member of the family of soluble defense collagens, negatively regulates the growth of myelomonocytic progenitors and the functions of macrophages. *Blood*.

[18] Turner JJO, Smolinska MJ, Sacre SM, Foxwell BMJ (2009). Induction of TLR tolerance in human macrophages by adiponectin: does LPS play a role?. *Scandinavian Journal of Immunology*.

[19] Ohashi K, Parker JL, Ouchi N (2010). Adiponectin promotes macrophage polarization toward an anti-inflammatory phenotype. *Journal of Biological Chemistry*.

[20] Holland WL, Miller RA, Wang ZV (2011). Receptor-mediated activation of ceramidase activity initiates the pleiotropic actions of adiponectin. *Nature Medicine*.

[21] Tong K-M, Chen C-P, Huang K-C (2011). Adiponectin increases MMP-3 expression in human chondrocytes through adipor1 signaling pathway. *Journal of Cellular Biochemistry*.

[22] Lago R, Gomez R, Otero M (2008). A new player in cartilage homeostasis: adiponectin induces nitric oxide synthase type II and pro-inflammatory cytokines in chondrocytes. *Osteoarthritis and Cartilage*.

[23] Gómez R, Scotece M, Conde J, Gómez-Reino JJ, Lago F, Gualillo O (2011). Adiponectin and leptin increase IL-8 production in human chondrocytes. *Annals of the Rheumatic Diseases*.

[24] Kusunoki N, Kitahara K, Kojima F (2010). Adiponectin stimulates prostaglandin E2 production in rheumatoid arthritis synovial fibroblasts. *Arthritis and Rheumatism*.

[25] Kitahara K, Kusunoki N, Kakiuchi T, Suguro T, Kawai S (2009). Adiponectin stimulates IL-8 production by rheumatoid synovial fibroblasts. *Biochemical and Biophysical Research Communications*.

[26] Frommer KW, Zimmermann B, Meier FMP (2010). Adiponectin-mediated changes in effector cells involved in the pathophysiology of rheumatoid arthritis. *Arthritis and Rheumatism*.

[27] Giles JT, Allison M, Bingham CO, Scott WM, Bathon JM (2009). Adiponectin is a mediator of the inverse association of adiposity with radiographic damage in rheumatoid arthritis. *Arthritis Care and Research*.

[28] Otero M, Logo R, Gomez R (2006). Changes in plasma levels of fat-derived hormones adiponectin, leptin, resistin and visfatin in patients with rheumatoid arthritis. *Annals of the Rheumatic Diseases*.

[29] Schäffler A, Ehling A, Neumann E (2003). Adipocytokines in synovial fluid. *Journal of the American Medical Association*.

[30] Chen X, Lu J, Bao J, Guo J, Shi J, Wang Y (2013). Adiponectin: a biomarker for rheumatoid arthritis?. *Cytokine & Growth Factor Reviews*.

[31] Klein-Wieringa IR, van der Linden MPM, Knevel R (2011). Baseline serum adipokine levels predict radiographic progression in early rheumatoid arthritis. *Arthritis and Rheumatism*.

[32] Nagashima T, Okubo-Fornbacher H, Aoki Y (2008). Increase in plasma levels of adiponectin after administration of anti-tumor necrosis factor agents in patients with rheumatoid arthritis. *Journal of Rheumatology*.

[33] Lewicki M, Kotyla P, Kucharz E (2009). Increased adiponectin levels in women with rheumatoid arthritis after etanercept treatment. *Journal of Rheumatology*.

[34] Frommer KW, Schaffler A, Buchler C (2012). Adiponectin isoforms: a potential therapeutic target in rheumatoid arthritis?. *Annals of the Rheumatic Diseases*.

[35] Zhang Y, Proenca R, Maffei M, Barone M, Leopold L, Friedman JM (1994). Positional cloning of the mouse obese gene and its human homologue. *Nature*.

[36] Ghilardi N, Skoda RC (1997). The leptin receptor activates janus kinase 2 and signals for proliferation in a factor-dependent cell line. *Molecular Endocrinology*.

[37] Frühbeck G (2006). Intracellular signalling pathways activated by leptin. *Biochemical Journal*.

[38] Ahlma RS, Prabakaran D, Mantzoros C (1996). Role of leptin in the neuroendocrine response to fasting. *Nature*.

[39] Jéquier E (2002). Leptin signaling, adiposity, and energy balance. *Annals of the New York Academy of Sciences*.

[40] Gualillo O, Eiras S, Lago F, Diéguez C, Casanueva FF (2000). Elevated serum leptin concentrations induced by experimental acute inflammation. *Life Sciences*.

[41] Santos-Alvarez J, Goberna R, Sánchez-Margalet V (1999). Human leptin stimulates proliferation and activation of human circulating monocytes. *Cellular Immunology*.

[42] Kiguchi N, Maeda T, Kobayashi Y, Fukazawa Y, Kishioka S (2009). Leptin enhances CC-chemokine ligand expression in cultured murine macrophage. *Biochemical and Biophysical Research Communications*.

[43] Lord GM, Matarese G, Howard JK, Baker RJ, Bloom SR, Lechler RI (1998). Leptin modulates the T-cell immune response and reverses starvation-induced immunosuppression. *Nature*.

[44] Faggioni R, Jones-Carson J, Reed DA (2000). Leptin-deficient (ob/ob) mice are protected from t cell-mediated hepatotoxicity: role of tumor necrosis factor *α* and IL-18. *Proceedings of the National Academy of Sciences of the United States of America*.

[45] Matarese G, di Giacomo A, Sanna V (2001). Requirement for leptin in the induction and progression of autoimmune encephalomyelitis. *Journal of Immunology*.

[46] Lee S-W, Park M-C, Park Y-B, Lee S-K (2007). Measurement of the serum leptin level could assist disease activity monitoring in rheumatoid arthritis. *Rheumatology International*.

[47] Yoshino T, Kusunoki N, Tanaka N (2011). Elevated serum levels of resistin, leptin, and adiponectin are associated with c-reactive protein and also other clinical conditions in rheumatoid arthritis. *Internal Medicine*.

[48] Hizmetli S, Kisa M, Gokalp N, Bakici MZ (2007). Are plasma and synovial fluid leptin levels correlated with disease activity in rheumatoid arthritis?. *Rheumatology International*.

[49] Busso N, So A, Chobaz-Péclat V (2002). Leptin signaling deficiency impairs humoral and cellular immune responses and attenuates experimental arthritis. *Journal of Immunology*.

[50] Bernotiene E, Palmer G, Gabay C (2006). The role of leptin in innate and adaptive immune responses. *Arthritis Research and Therapy*.

[51] Hultgren OH, Tarkowski A (2001). Leptin in septic arthritis: decreased levels during infection and amelioration of disease activity upon its adminstration. *Arthritis Research*.

[52] Derdemezis CS, Filippatos TD, Voulgari PV, Tselepis AD, Drosos AA, Kiortsis DN (2009). Effects of a 6-month infliximab treatment on plasma levels of leptin and adiponectin in patients with rheumatoid arthritis. *Fundamental and Clinical Pharmacology*.

[53] Gonzalez-Gay MA, Garcia-Unzueta MT, Berja A (2009). Anti-TNF-*α* therapy does not modulate leptin in patients with severe rheumatoid arthritis. *Clinical and Experimental Rheumatology*.

[54] Engvall I-L, Tengstrand B, Brismar K, Hafström I (2010). Infliximab therapy increases body fat mass in early rheumatoid arthritis independently of changes in disease activity and levels of leptin and adiponectin: a randomised study over 21 months. *Arthritis Research and Therapy*.

[55] Olama SM, Senna MK, Elarman M (2012). Synovial/Serum leptin ratio in rheumatoid arthritis: the association with activity and erosion. *Rheumatology International*.

[56] Otero M, Lago R, Lago F, Reino JJG, Gualillo O (2005). Signalling pathway involved in nitric oxide synthase type II activation in chondrocytes: synergistic effect of leptin with interleukin-1. *Arthritis Research & Therapy*.

[57] Otero M, Lago R, Gómez R, Lago F, Gomez-Reino JJ, Gualillo O (2007). Phosphatidylinositol 3-kinase, MEK-1 and p38 mediate leptin/interferon-gamma synergistic NOS type II induction in chondrocytes. *Life Sciences*.

[58] Bao J-P, Chen W-P, Feng J, Hu P-F, Shi Z-L, Wu L-D (2010). Leptin plays a catabolic role on articular cartilage. *Molecular Biology Reports*.

[59] Tong K-M, Shieh D-C, Chen C-P (2008). Leptin induces IL-8 expression via leptin receptor, IRS-1, PI3K, Akt cascade and promotion of NF-*κ*B/p300 binding in human synovial fibroblasts. *Cellular Signalling*.

[60] Lam QLK, Lu L (2007). Role of leptin in immunity. *Cellular & Molecular Immunology*.

[61] Lam QLK, Zheng B-J, Jin D-Y, Cao X, Lu L (2007). Leptin induces CD40 expression through the activation of Akt in murine dendritic cells. *Journal of Biological Chemistry*.

[62] Matarese G, Carrieri PB, La Cava A (2005). Leptin increase in multiple sclerosis associates with reduced number of CD4^+^CD25^+^ regulatory T cells. *Proceedings of the National Academy of Sciences of the United States of America*.

[63] de Rosa V, Procaccini C, Calì G (2007). A key role of leptin in the control of regulatory T cell proliferation. *Immunity*.

[64] Notley CA, Ehrenstein MR (2010). The yin and yang of regulatory T cells and inflammation in RA. *Nature Reviews Rheumatology*.

[65] Procaccini C, de Rosa V, Galgani M (2010). An oscillatory switch in mTOR kinase activity sets regulatory T cell responsiveness. *Immunity*.

[66] Nagpal S, Patel S, Jacobe H (1997). Tazarotene-induced gene 2 (TIG2), a novel retinoid-responsive gene in skin. *Journal of Investigative Dermatology*.

[67] Wittamer V, Franssen J-D, Vulcano M (2003). Specific recruitment of antigen-presenting cells by chemerin, a novel processed ligand from human inflammatory fluids. *Journal of Experimental Medicine*.

[68] Wittamer V, Grégoire F, Robberecht P, Vassart G, Communi D, Parmentier M (2004). The C-terminal nonapeptide of mature chemerin activates the chemerin receptor with low nanomolar potency. *Journal of Biological Chemistry*.

[69] Vermi W, Riboldi E, Wittamer V (2005). Role of ChemR23 in directing the migration of myeloid and plasmacytoid dendritic cells to lymphoid organs and inflamed skin. *Journal of Experimental Medicine*.

[70] Parolini S, Santoro A, Marcenaro E (2007). The role of chemerin in the colocalization of NK and dendritic cell subsets into inflamed tissues. *Blood*.

[71] Zabel BA, Ohyama T, Zuniga L (2006). Chemokine-like receptor 1 expression by macrophages in vivo: regulation by TGF-*β* and TLR ligands. *Experimental Hematology*.

[72] Albanesi C, Scarponi C, Pallotta S (2009). Chemerin expression marks early psoriatic skin lesions and correlates with plasmacytoid dendritic cell recruitment. *Journal of Experimental Medicine*.

[73] Parlee SD, Ernst MC, Muruganandan S, Sinal CJ, Goralski KB (2010). Serum chemerin levels vary with time of day and are modified by obesity and tumor necrosis factor-*α*. *Endocrinology*.

[74] Bozaoglu K, Bolton K, McMillan J (2007). Chemerin is a novel adipokine associated with obesity and metabolic syndrome. *Endocrinology*.

[75] Lehrke M, Becker A, Greif M (2009). Chemerin is associated with markers of inflammation and components of the metabolic syndrome but does not predict coronary atherosclerosis. *European Journal of Endocrinology*.

[76] Jialal I, Devaraj S, Kaur H, Adams-Huet B, Bremer AA (2013). Increased chemerin and decreased omentin-1 in both adipose tissue and plasma in nascent metabolic syndrome. *Journal of Clinical Endocrinology and Metabolism*.

[77] Ernst MC, Haidl ID, Zuńĩga LA (2012). Disruption of the chemokine-like receptor-1 (CMKLR1) gene is associated with reduced adiposity and glucose intolerance. *Endocrinology*.

[78] Berg V, Sveinbjörnsson B, Bendiksen S, Brox J, Meknas K, Figenschau Y (2010). Human articular chondrocytes express ChemR23 and chemerin; ChemR23 promotes inflammatory signalling upon binding the ligand chemerin21-157. *Arthritis Research and Therapy*.

[79] Kaneko K, Miyabe Y, Takayasu A (2011). Chemerin activates fibroblast-like synoviocytes in patients with rheumatoid arthritis. *Arthritis Research and Therapy*.

[80] Ha YJ, Kang EJ, Song JS, Park YB, Lee SK, Choi ST (2013). Plasma chemerin levels in rheumatoid arthritis are correlated with disease activity rather than obesity. *Joint Bone Spine*.

[81] Herenius MM, Oliveira AS, Wijbrandts CA, Gerlag DM, Tak PP, Lebre MC (2013). Anti-TNF therapy reduces serum levels of chemerin in rheumatoid arthritis: a new mechanism by which anti-TNF might reduce inflammation. *PLoS ONE*.

[82] Samal B, Sun Y, Stearns G, Xie C, Suggs S, McNiece I (1994). Cloning and characterization of the cDNA encoding a novel human pre-B- cell colony-enhancing factor. *Molecular and Cellular Biology*.

[83] Friebe D, Neef M, Kratzsch J (2011). Leucocytes are a major source of circulating nicotinamide phosphoribosyltransferase (NAMPT)/pre-B cell colony (PBEF)/visfatin linking obesity and inflammation in humans. *Diabetologia*.

[84] Catalán V, Gómez-Ambrosi J, Rodríguez A (2011). Association of increased Visfatin/PBEF/NAMPT circulating concentrations and gene expression levels in peripheral blood cells with lipid metabolism and fatty liver in human morbid obesity. *Nutrition, Metabolism and Cardiovascular Diseases*.

[85] Curat CA, Wegner V, Sengenès C (2006). Macrophages in human visceral adipose tissue: increased accumulation in obesity and a source of resistin and visfatin. *Diabetologia*.

[86] Jia SH, Li Y, Parodo J (2004). Pre-B cell colony-enhancing factor inhibits neutrophil apoptosis in experimental inflammation and clinical sepsis. *Journal of Clinical Investigation*.

[87] Fukuhara A, Matsuda M, Nishizawa M (2005). Visfatin: a protein secreted by visceral fat that mimics the effects of insulin. *Science*.

[88] Fukuhara A, Matsuda M, Nishizawa M (2007). Erratum (Retracted article): Visfatin: a protein secreted by visceral fat that mimics the effects of insulin. *Science*.

[89] Jacques C, Holzenberger M, Mladenovic Z (2012). Proinflammatory actions of visfatin/nicotinamide phosphoribosyltransferase (Nampt) involve regulation of insulin signaling pathway and Nampt enzymatic activity. *Journal of Biological Chemistry*.

[90] Haider DG, Schindler K, Schaller G, Prager G, Wolzt M, Ludvik B (2006). Increased plasma visfatin concentrations in morbidly obese subjects are reduced after gastric banding. *Journal of Clinical Endocrinology and Metabolism*.

[91] Moschen AR, Kaser A, Enrich B (2007). Visfatin, an adipocytokine with proinflammatory and immunomodulating properties. *Journal of Immunology*.

[92] Lee W-J, Wu C-S, Lin H (2009). Visfatin-induced expression of inflammatory mediators in human endothelial cells through the NF-B pathway. *International Journal of Obesity*.

[93] Dahl TB, Yndestad A, Skjelland M (2007). Increased expression of visfatin in macrophages of human unstable carotid and coronary atherosclerosis: possible role in inflammation and plaque destabilization. *Circulation*.

[94] Nowell MA, Richards PJ, Fielding CA (2006). Regulation of pre-B cell colony-enhancing factor by STAT-3-dependent interleukin-6 trans-signaling: implications in the pathogenesis of rheumatoid arthritis. *Arthritis and Rheumatism*.

[95] Brentano F, Schorr O, Ospelt C (2007). Pre-B cell colony-enhancing factor/visfatin, a new marker of inflammation in rheumatoid arthritis with proinflammatory and matrix-degrading activities. *Arthritis and Rheumatism*.

[96] Matsui H, Tsutsumi A, Sugihara M (2008). Visfatin (pre-B cell colony-enhancing factor) gene expression in patients with rheumatoid arthritis. *Annals of the Rheumatic Diseases*.

[97] Meier FM, Frommer KW, Peters MA (2012). Visfatin/pre-B-cell colony-enhancing factor (PBEF), a proinflammatory and cell motility-changing factor in rheumatoid arthritis. *Journal of Biological Chemistry*.

[98] Evans L, Williams AS, Hayes AJ, Jones SA, Nowell M (2011). Suppression of leukocyte infiltration and cartilage degradation by selective inhibition of pre-B cell colony-enhancing factor/visfatin/nicotinamide phosphoribosyltransferase: apo866-mediated therapy in human fibroblasts and murine collagen-induced arthritis. *Arthritis and Rheumatism*.

[99] Busso N, Karababa M, Nobile M (2008). Pharmacological inhibition of nicotinamide phosphoribosyltransferase/visfatin enzymatic activity identifies a new inflammatory pathway linked to NAD. *PLoS ONE*.

[100]  Rho YH, Solus J, Sokka T (2009). Adipocytokines are associated with radiographic joint damage in rheumatoid arthritis. *Arthritis and Rheumatism*.

[101] Gonzalez-Gay MA, Vazquez-Rodriguez TR, Garcia-Unzueta MT (2010). Visfatin is not associated with inflammation or metabolic syndrome in patients with severe rheumatoid arthritis undergoing anti-TNF-*α* therapy. *Clinical and Experimental Rheumatology*.

[102] Šenolt L, Kryštůfková O, Hulejová H (2011). The level of serum visfatin (PBEF) is associated with total number of B cells in patients with rheumatoid arthritis and decreases following B cell depletion therapy. *Cytokine*.

[103] Steppan CM, Bailey ST, Bhat S (2001). The hormone resistin links obesity to diabetes. *Nature*.

[104] Heilbronn LK, Rood J, Janderova L (2004). Relationship between serum resistin concentrations and insulin resistance in nonobese, obese, and obese diabetic subjects. *Journal of Clinical Endocrinology and Metabolism*.

[105] Lee JH, Chan JL, Yiannakouris N (2003). Circulating resistin levels are not associated with obesity or insulin resistance in humans and are not regulated by fasting or leptin administration: cross-sectional and interventional studies in normal, insulin-resistant, and diabetic subjects. *Journal of Clinical Endocrinology and Metabolism*.

[106] Tarkowski A, Bjersing J, Shestakov A, Bokarewa MI (2010). Resistin competes with lipopolysaccharide for binding to toll-like receptor 4. *Journal of Cellular and Molecular Medicine*.

[107] Son YM, Ahn SM, Kim GR (2010). Resistin enhances the expansion of regulatory T cells through modulation of dendritic cells. *BMC Immunology*.

[108] Kaser S, Kaser A, Sandhofer A, Ebenbichler CF, Tilg H, Patsch JR (2003). Resistin messenger-RNA expression is increased by proinflammatory cytokines in vitro. *Biochemical and Biophysical Research Communications*.

[109] Silswal N, Singh AK, Aruna B, Mukhopadhyay S, Ghosh S, Ehtesham NZ (2005). Human resistin stimulates the pro-inflammatory cytokines TNF-*α* and IL-12 in macrophages by NF-*κ*B-dependent pathway. *Biochemical and Biophysical Research Communications*.

[110] Bokarewa M, Nagaev I, Dahlberg L, Smith U, Tarkowski A (2005). Resistin, an adipokine with potent proinflammatory properties. *Journal of Immunology*.

[111] Fadda SM, Gamal SM, Elsaid NY, Mohy AM (2013). Resistin in inflammatory and degenerative rheumatologic diseases: relationship between resistin and rheumatoid arthritis disease progression. *Zeitschrift für Rheumatologie*.

[112] Šenolt L, Housa D, Vernerová Z (2007). Resistin in rheumatoid arthritis synovial tissue, synovial fluid and serum. *Annals of the Rheumatic Diseases*.

[113] Migita K, Maeda Y, Miyashita T (2006). The serum levels of resistin in rheumatoid arthritis patients. *Clinical and Experimental Rheumatology*.

[114] Forsblad d’elia H, Pullerits R, Carlsten H, Bokarewa M (2008). Resistin in serum is associated with higher levels of IL-1Ra in post-menopausal women with rheumatoid arthritis. *Rheumatology*.

[115] Kontunen P, Vuolteenaho K, Nieminen R (2011). Resistin is linked to inflammation, and leptin to metabolic syndrome, in women with inflammatory arthritis. *Scandinavian Journal of Rheumatology*.

[116] Straburzyńska-Lupa A, Nowak A, Pilaczyńska-Szcześniak Ł (2010). Visfatin, resistin, hsCRP and insulin resistance in relation to abdominal obesity in women with rheumatoid arthritis. *Clinical and Experimental Rheumatology*.

[117] Alkady EAM, Ahmed HM, Tag L, Abdou MA (2011). Serum and synovial adiponectin, resistin, and visfatin levels in rheumatoid arthritis patients. Relation to disease activity. *Zeitschrift für Rheumatologie*.

[118] Gonzalez-Gay MA, Garcia-Unzueta MT, Gonzalez-Juanatey C (2008). Anti-TNF-*α* therapy modulates resistin in patients with rheumatoid arthritis. *Clinical and Experimental Rheumatology*.

[119] Klaasen R, Herenius MMJ, Wijbrandts CA (2012). Treatment-specific changes in circulating adipocytokines: a comparison between tumour necrosis factor blockade and glucocorticoid treatment for rheumatoid arthritis. *Annals of the Rheumatic Diseases*.

[120] Triebel S, Blaser J, Reinke H, Tschesche H (1992). A 25 kDa *α*2-microglobulin-related protein is a component of the 125 kDa form of human gelatinase. *FEBS Letters*.

[121] Borregaard N, Cowland JB (2006). Neutrophil gelatinase-associated lipocalin, a siderophore-binding eukaryotic protein. *BioMetals*.

[122] Chakraborty S, Kaur S, Guha S, Batra SK (2012). The multifaceted roles of neutrophil gelatinase associated lipocalin (NGAL) in inflammation and cancer. *Biochimica et Biophysica Acta. Reviews on Cancer*.

[123] Hvidberg V, Jacobsen C, Strong RK, Cowland JB, Moestrup SK, Borregaard N (2005). The endocytic receptor megalin binds the iron transporting neutrophil-gelatinase-associated lipocalin with high affinity and mediates its cellular uptake. *FEBS Letters*.

[124] Flower DR (1996). The lipocalin protein family: structure and function. *Biochemical Journal*.

[125] Chu S-T, Lin H-J, Huang H-L, Chen Y-H (1998). The hydrophobic pocket of 24p3 protein from mouse uterine luminal fluid: fatty acid and retinol binding activity and predicted structural similarity to lipocalins. *Journal of Peptide Research*.

[126] Yang J, Goetz D, Li J-Y (2002). An iron delivery pathway mediated by a lipocalin. *Molecular Cell*.

[127] Liu Z, Yang A, Wang Z (2011). Multiple apoptotic defects in hematopoietic cells from mice lacking lipocalin 24p3. *Journal of Biological Chemistry*.

[128] Goetz DH, Holmes MA, Borregaard N, Bluhm ME, Raymond KN, Strong RK (2002). The neutrophil lipocalin NGAL is a bacteriostatic agent that interferes with siderophore-mediated iron acquisition. *Molecular Cell*.

[129] Liu Z, Petersen R, Devireddy L (2013). Impaired neutrophil function in 24p3 null mice contributes to enhanced susceptibility to bacterial infections. *Journal of Immunology*.

[130] Jiang W, Constante M, Santos MM (2008). Anemia upregulates lipocalin 2 in the liver and serum. *Blood Cells, Molecules, and Diseases*.

[131] Yan Q-W, Yang Q, Mody N (2007). The adipokine lipocalin 2 is regulated by obesity and promotes insulin resistance. *Diabetes*.

[132] Cowland JB, Muta T, Borregaard N (2006). IL-1*β*-specific up-regulation of neutrophil gelatinase-associated lipocalin is controlled by I*κ*B-*ζ*. *Journal of Immunology*.

[133] Owen HC, Roberts SJ, Ahmed SF, Farquharson C (2008). Dexamethasone-induced expression of the glucocorticoid response gene lipocalin 2 in chondrocytes. *American Journal of Physiology. Endocrinology and Metabolism*.

[134] Conde J, Gomez R, Bianco G (2011). Expanding the adipokine network in cartilage: identification and regulation of novel factors in human and murine chondrocytes. *Annals of the Rheumatic Diseases*.

[135] Tschesche H, Zölzer V, Triebel S, Bartsch S (2001). The human neutrophil lipocalin supports the allosteric activation of matrix metalloproteinases. *European Journal of Biochemistry*.

[136] Gruber BL, Sorbi D, French DL (1996). Markedly elevated serum MMP-9 (Gelatinase B) levels in rheumatoid arthritis: a potentially useful laboratory marker. *Clinical Immunology and Immunopathology*.

[137] Katano M, Okamoto K, Arito M (2009). Implication of granulocyte-macrophage colony-stimulating factor induced neutrophil gelatinase-associated lipocalin in pathogenesis of rheumatoid arthritis revealed by proteome analysis. *Arthritis Research and Therapy*.

[138] Uhlar CM, Whitehead AS (1999). Serum amyloid A, the major vertebrate acute-phase reactant. *European Journal of Biochemistry*.

[139] Reigstad CS, Lundén GÖ, Felin J, Bäckhed F (2009). Regulation of serum amyloid A3 (SAA3) in mouse colonic epithelium and adipose tissue by the intestinal microbiota. *PLoS ONE*.

[140] Frasshauer M, Klein J, Kralisch S (2004). Serum amyloid A3 expression is stimulated by dexamethasone and interleukin-6 in 3T3-L1 adipocytes. *Journal of Endocrinology*.

[141] Lin Y, Rajala MW, Berger JP, Moller DE, Barzilai N, Scherer PE (2001). Hyperglycemia-induced production of acute phase reactants in adipose tissue. *Journal of Biological Chemistry*.

[142] Chang YH, Subramanian S, Chan CK (2007). Adipocyte-derived serum amyloid A3 and hyaluronan play a role in monocyte recruitment and adhesion. *Diabetes*.

[143] Son D-S, Arai KY, Roby KF, Terranova PF (2004). Tumor Necrosis Factor *α* (TNF) increases granulosa cell proliferation: dependence on c-Jun and TNF receptor type 1. *Endocrinology*.

[144] Deguchi A, Tomita T, Omori T (2013). Serum amyloid A3 binds MD-2 to activate p38 and NF-*κ*B pathways in a MyD88-dependent manner. *Journal of Immunology*.

[145] Vallon R, Freuler F, Desta-Tsedu N (2001). Serum amyloid a (apoSAA) expression is up-regulated in rheumatoid arthritis and induces transcription of matrix metalloproteinases. *Journal of Immunology*.

[146] Geurts J, Vermeij EA, Pohlers D (2011). A novel Saa3-promoter reporter distinguishes inflammatory subtypes in experimental arthritis and human synovial fibroblasts. *Annals of the Rheumatic Diseases*.

[147] Hida K, Wada J, Eguchi J (2005). Visceral adipose tissue-derived serine protease inhibitor: a unique insulin-sensitizing adipocytokine in obesity. *Proceedings of the National Academy of Sciences of the United States of America*.

[148] Klöting N, Berndt J, Kralisch S (2006). Vaspin gene expression in human adipose tissue: association with obesity and type 2 diabetes. *Biochemical and Biophysical Research Communications*.

[149] Ozgen M, Koca SS, Dagli N, Balin M, Ustundag B, Isik A (2010). Serum adiponectin and vaspin levels in rheumatoid arthritis. *Archives of Medical Research*.

[150] Šenolt L, Polanská M, Filková M (2010). Vaspin and omentin: new adipokines differentially regulated at the site of inflammation in rheumatoid arthritis. *Annals of the Rheumatic Diseases*.

[151] Schäffler A, Neumeier M, Herfarth H, Fürst A, Schölmerich J, Büchler C (2005). Genomic structure of human omentin, a new adipocytokine expressed in omental adipose tissue. *Biochimica et Biophysica Acta. Gene Structure and Expression*.

[152] de Souza Batista CM, Yang R-Z, Lee M-J (2007). Omentin plasma levels and gene expression are decreased in obesity. *Diabetes*.

[153] Cantarini L, Simonini G, Fioravanti A (2011). Circulating levels of the adipokines vaspin and omentin in patients with juvenile idiopathic arthritis, and relation to disease activity. *Clinical and Experimental Rheumatology*.

[154] Tatemoto K, Hosoya M, Habata Y (1998). Isolation and characterization of a novel endogenous peptide ligand for the human APJ receptor. *Biochemical and Biophysical Research Communications*.

[155] Boucher J, Masri B, Daviaud D (2005). Apelin, a newly identified adipokine up-regulated by insulin and obesity. *Endocrinology*.

[156] Daviaud D, Boucher J, Gesta S (2006). TNFalpha up-regulates apelin expression in human and mouse adipose tissue. *The FASEB Journal*.

[157] Hu P-F, Tang J-L, Chen W-P, Bao J-P, Wu L-D (2011). Increased apelin serum levels and expression in human chondrocytes in osteoarthritic patients. *International Orthopaedics*.

[158] di Franco M, Spinelli FR, Metere A (2012). Serum levels of asymmetric dimethylarginine and apelin as potential markers of vascular endothelial dysfunction in early rheumatoid arthritis. *Mediators of Inflammation*.

[159] White RT, Damm D, Hancock N (1992). Human adipsin is identical to complement factor D and is expressed at high levels in adipose tissue. *Journal of Biological Chemistry*.

[160] Vuolteenaho K, Hannonen P, Kauppi M (2010). Predictive value of pretretment adipocytokine levels for remission rates in early RA treated with DMARDS and infliximab. *Basic & Clinical Pharmacology & Toxicology*.

